# Pregnancy-associated melanoma: characteristics and outcomes from 2002 to 2020

**DOI:** 10.1097/CMR.0000000000000953

**Published:** 2024-01-23

**Authors:** Tara M. Davidson, Tina J. Hieken, Amy E. Glasgow, Elizabeth B. Habermann, Yiyi Yan

**Affiliations:** a Department of Internal Medicine, Mayo Clinic; b Division of Breast and Melanoma Surgical Oncology, Department of Surgery, Mayo Clinic; c Robert D. and Patricia E. Kern Center for the Science of Health Care Delivery, Mayo Clinic; d Division of Health Care Delivery Research, Mayo Clinic; e Department of Medical Oncology, Mayo Clinic, Rochester, Minnesota, USA

**Keywords:** melanoma, pregnancy-associated malignancy, pregnancy-associated melanoma

## Abstract

Melanoma diagnosed within 1 year of pregnancy is defined as pregnancy-associated melanoma (PAM). No robust data on how pregnancy influences melanoma nor guidelines for PAM management exist. With IRB approval, female patients with a pathology-confirmed melanoma diagnosis within 1 year of pregnancy treated at our institution from 2000 to 2020 were identified. Controls from the cancer registry were matched 1 : 4 when available on decade of age, year of surgery (±5), and stage. We identified 83 PAM patients with median follow-up of 86 months. Mean age at diagnosis was 31 years. 80% AJCC V8 stage I, 2.4% stage II, 13% stage III, 4.8% stage IV. Mean Breslow thickness was 0.79 mm and 3.6% exhibited ulceration. The mean mitotic rate was 0.76/mm^2^. In terms of PAM management, 98.6% of ESD patients and 86.7% of LSD patients received standard-of-care therapy per NCCN guidelines for their disease stage. No clinically significant delays in treatment were noted. Time to treatment from diagnosis to systemic therapy for LSD patients was an average of 46 days (95% CI: 34–59 days). Comparing the 83 PAM patients to 309 controls matched on age, stage, and year of diagnosis, similar 5-year overall survival (97% vs. 97%, *P* = 0.95) or recurrence-free survival (96% vs. 96%, *P* = 0.86) was observed. The outcomes of PAM following SOC treatment at a highly specialized center for melanoma care were comparable to non-PAM when matched by clinical-pathologic features. Specialty center care is encouraged for women with PAM.

## Introduction

Melanoma is one of the most common cancers in young females, and one-third of all cases of melanoma in females are diagnosed during childbearing years [1,2]. Melanoma is also the most common malignancy in pregnancy, accounting for 31% of all cancers identified during gestation with incidence rates increasing worldwide [3]. This study investigates pregnancy-associated melanoma (PAM), defined as melanoma diagnosed within 1 year prior to conception date and up to 1 year following birth or fetal loss [4].

Dating back to the 1950s, there have been concerns about pregnancy’s effect on melanoma prognosis. It is well known that increased melanocytic activity and hyperpigmentation is commonly observed during pregnancy, with clinical findings such as the linea nigra, melasma, and genital/areolar darkening. In addition, there are changes in benign melanocytic nevi which occur during pregnancy (or with stimulation by progesterone) including increased mitotic counts, diameter growth (most notable in areas of skin stretching), increased vascularity and a histologic pattern of amplified melanocyte clustering in the superficial dermis of nevi [5–8]. These findings led to the presumption that pregnancy must also increase the rate of malignant nevi transformation. Clinical and histologic data has proven this assumption untrue with resolution of a majority of these benign changes within 12 months postpartum [5,7–9]. To biologically confirm this assumption, Facina *et al*. used mice models to assess the expression of apoptosis-related genes in melanoma tumors during pregnancy and ultimately concluded that pregnancy activated intrinsic apoptosis to stimulate *caspases 7* and *9*, but that the overall net result was inhibition of apoptosis mechanisms. Ultimately, they concluded that pregnancy was not felt to worsen melanoma in mice [10]. Pregnancy, however, has been shown to increase the degree of atypia in dysplastic nevi with some speculation that increased estrogen beta receptor expression as the driver of these changes [9,11]. Interestingly, anti-estrogen drugs, like tamoxifen, also result in an increase of estrogen beta receptor activity with no associated increased risk of melanoma [12]. Instead, the benign nevus changes seen in pregnancy are likely due to more complex processes. Despite the number of women affected with peripartum melanoma, there is a lack of robust data on PAM, with existing reports presenting conflicting results and few with granular data or long-term follow-up on large-scale patient cohorts. Further, the definition of PAM itself varies within publications, ranging from 1 year prior to up to 2 years following delivery. Several studies have showed PAM to present at increased depth, and be associated with higher metastatic risk and worse outcomes compared to melanoma [13–16]. However, a number of other studies directly contradict these findings and suggest that outcomes for PAM are not worse than for non-PAM patients, regardless of stage [17–19]. We define PAM as melanoma diagnosed within 1 year prior to conception date and up to 1 year following birth or fetal loss. We used the year prior to pregnancy to a year after pregnancy definition for PAM to capture patients felt to be most vulnerable for melanoma recurrence or initial PAM development secondary to the hormonal changes associated with pregnancy.

Additional studies are critically needed to deepen our knowledge and guide management of this clinically challenging situation. Clarity regarding PAM characteristics and outcomes can help formulate treatment guidelines which are especially important in the era of targeted and immunotherapy. Our study addresses this important clinical knowledge gap.

## Methods

With IRB approval, female patients with a pathology-confirmed melanoma diagnosed within 1 year of pregnancy and treated at our institution from 2000 to 2020 were identified using ICD9 and 10 diagnosis codes. We used the medical definition of pregnancy – the period in which a fetus develops inside a woman’s uterus and included patients who had miscarriage, abortions, or live births. We used the dates of conception within the medical record to define the date of pregnancy for our patients. Study team members confirmed patients met inclusion criteria through manual review. Patients who refused use of their records for research were excluded per State of Minnesota law. Stage was calculated using the American Joint Commission for Cancer, 8th edition staging criteria. We defined stage I and II melanoma as early-stage disease (ESD) and stage III and IV as late-stage disease (LSD).

Potential controls, cases of melanoma from 2000-2020 in women aged 18–50 years, were identified from our institutional cancer registry. Controls were matched 1 : 4 when available on decade of age, year of surgery (±5), and stage of disease.

Survival and time to recurrence were analyzed using the Kaplan–Meier method and compared using the log-rank test. Patients were censored at time of death or loss to follow-up. All analysis was performed with SAS software (version 9.4, SAS Institute Inc., Cary, NC), and *P*-values of <0.05 were considered statistically significant.

## Results

We identified 83 PAM patients meeting our inclusion criteria. Patient, tumor, and pregnancy details are summarized in Tables 1 and 2. Mean age at diagnosis was 31 years with most patients (77%) between the ages of 26 and 35. Mean BMI at melanoma diagnosis was 21.8 kg/m^2^ (healthy weight). Twenty patients (24%) were diagnosed with melanoma within 1 year prior to their pregnancy, 30 (36%) during pregnancy and 33 (40%) were diagnosed within the year after pregnancy. Of those patients who were pregnant at the time of diagnosis, 12 of 30 (60%) were in the first trimester, 10 (33%) were in the second trimester and 8 (27%) were in the third trimester. Mean gravidity (G, defined as the number of times that a woman has been pregnant) at the time of PAM diagnosis was 1.9 pregnancies, with most women G1 (25%) and G2 (31%). 12% of patients were ≥ G3 and in 9.6% the number of pregnancies was unknown. Mean parity (P, defined as the number of times that she has given birth to a fetus with a gestational age of 24 weeks or more, regardless of whether the child was born alive or was stillborn) at the time of PAM diagnosis was 1.2 with the majority P1 (39%) and P2 (24%).

**Table 1:** Patient and tumor characteristics

N	83
Mean months from diagnosis to last contact	100.2
Deceased at last contact	4 (4.8%)
Mean age at diagnosis	31.1
Age <26	7 (8.4%)
Age 26–35	64 (77.1%)
Age >35	12 (14.5%)
Race	
White	83 (100%)
Mean BMI at PAM diagnosis	21.8
Stage at PAM diagnosis	
I (ESD)	66 (79.5%)
TIA	57 (68.7%)
TIB	9 (10.8%)
II (ESD)	2 (2.4%)
TIIA	2 (2.4%)
III (LSD)	11 (13.3%)
IV (LSD)	4 (4.8%)
Mean Breslow depth	0.79
≤ 0.8	55 (66.3%)
0.8–<2.00	15 (18.1%)
2.00–≤4.00	5 (6.0%)
>4.00	1 (1.2%)
N/A	7 (8.4%)
Positive for ulceration at diagnosis	3 (3.6%)
Mean mitotic rate	0.76
0 mit/mm^2^	44 (53.0%)
1 mit/mm^2^	6 (7.2%)
≥1 mit/mm^2^	12 (14.5%)
Not reported	21 (25.3%)
Late-stage pN category (n = 15)	
p0	3 (20%)
pN1	9 (60.0%)
pN2	2 (13.3%)
pN3	1 (6.7%)
Melanoma location	
Head/Neck	6 (7.2%)
Trunk	42 (50.6%)
Upper extremity	8 (9.6%)
Lower extremity	27 (32.5%)

**Table 2:** Pregnancy characteristics

Melanoma diagnosis relative to pregnancy	
Before	20 (24.1%)
During	31 (37.3%)
First trimester	12
Second trimester	11
Third trimester	8
After	32 (38.6%)
Gravity at diagnosis	
Mean gravity	1.9
G0	9 (10.8%)
G1	21 (25.3%)
G2	26 (31.3%)
G3	9 (10.8%)
>G3	10 (12.0%)
Unknown	8 (9.6%)
Parity at diagnosis	
Mean parity	1.2
P0	17 (20.5%)
P1	32 (38.6%)
P2	20 (24.1%)
>P3	6 (7.2%)
Unknown	8 (9.6%)

In terms of PAM characteristics, there were 68 patients with ESD and 15 patients with LSD. Specifically, 70.5% were stage I, 2.4% were stage II, 13.3% were stage III, and 4.8% were stage IV at diagnosis. Mean Breslow thickness of the primary melanoma was 0.79 mm, with 66.3% of tumors ≤ 0.8 mm, 18.1% between 0.81 mm and 2 mm, 7.2% were > 2 mm and 8.4% had unreported Breslow depth. Only 3.6% of tumors were noted to be ulcerated. Mean tumor mitotic rate was 0.76/mm^2^, with 53% having a mitotic rate of 0 mit/mm^2^, 7.2% 1 mit/mm^2^, 15% ≥ 1 mit/mm^2^, and 25% mitotic rate unreported. The anatomic site of the index melanoma was head/neck in 7.2% (1 uveal melanoma), trunk in 51%, lower extremity in 33% (6 acral melanomas) and upper extremity 9.6%. For the LSD patients, the nodal pathologic stage was pN0 in 30% (including 1 uveal melanoma patient, pN1 in 60%, pN2 in 13% and pN3 in 6.7%.

In terms of PAM management, 99% of ESD patients and 87% of LSD patients received standard-of-care (SOC) therapy including wide local excision (WLE), sentinal lymph node biopsy (SLNB), completion lymph node dissection (CLND) and adjuvant or systemic treatments [including BRAF-targeted therapy (TT) and/or immunotherapy] per NCCN Guidelines for their disease stage at the time of their diagnosis. One ESD patient refused the recommended SLNB. Two LSD patients chose observation over recommended adjuvant therapy. No clinically significant delays in treatment were noted, 2 ESD patients postponed WLE for 2 and 4 weeks respectively so it could be completed after delivery. Time to treatment from diagnosis to systemic therapy for late-stage patients was an average of 46 days (95% CI: 34–59 days). Two late-stage patients underwent early induction of labor, near 32 weeks to start systemic therapy and neither had poor delivery outcomes. These patients both received consultation with the high-risk maternal-fetal medicine team at our institution who were instrumental in the care of both baby and mother. Nine of 26 patients (35%) who underwent SLNB were SLN-positive and non-SLN metastases were seen in 2 of 7 (29%) who proceeding to CLND, 2 patients were not recommended CLND.

Our entire PAM cohort had a median follow-up time of 86 months (7.2 years), with a 5-year survival rate of 97%. Four patients overall were deceased at the time of follow-up: 2 died from progression of melanoma and 2 died from other causes unrelated to melanoma. 5-year recurrence free survival was 96%.

Exploring these cohorts in greater detail, ESD PAM patients had a median follow-up time of 8 years with a 5-year survival rate of 100%. Recurrence-free survival at 5 years was 98%. The only recurrence was locoregional. LSD patients had a median follow-up time of 4 years with an estimated 5-year survival rate of 82%. Relapse-free survival at 5 years was 85%. Two patients developed distant metastasis.

**Fig. 1:**
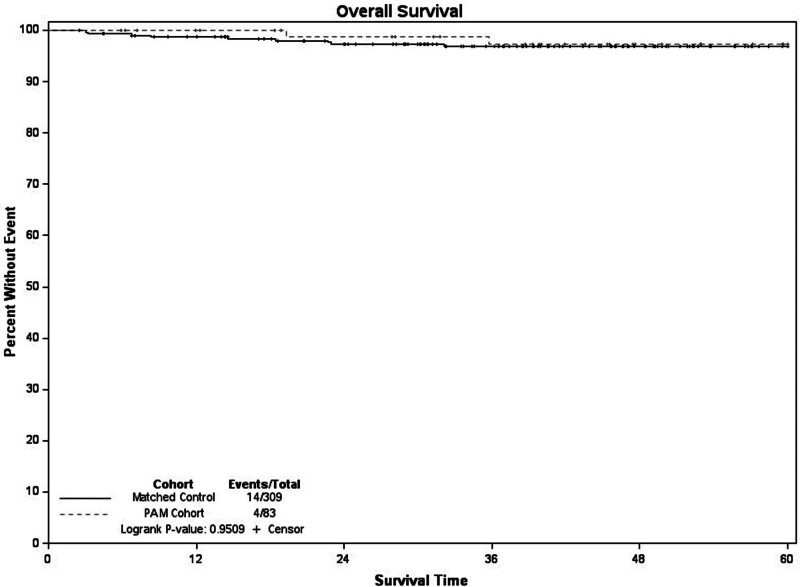
Overall survival PAM vs. non-PAM matched cohort.

**Fig. 2:**
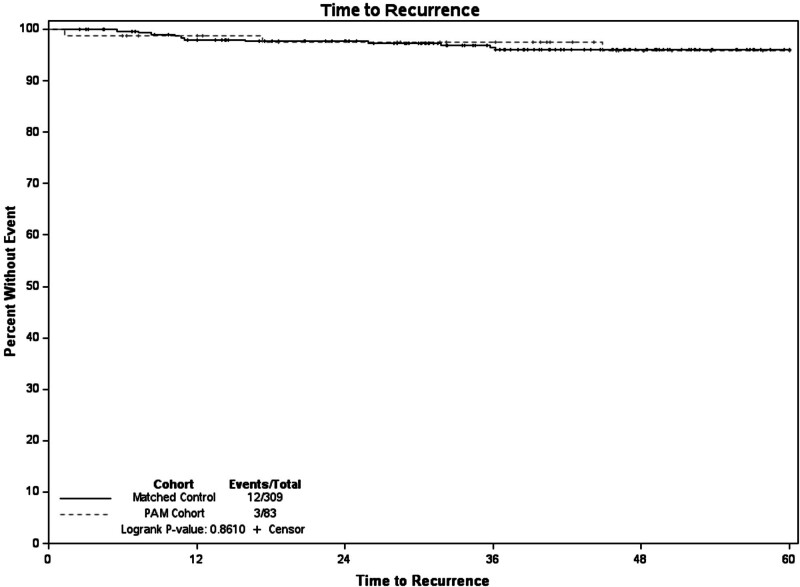
Time to recurrence PAM vs. non-PAM matched cohort.

When compared the 309 controls from our institutional cancer registry were matched on age decade, stage, and year of diagnosis (± 5 years) to our 83 PAM patients. Within the matched cohort 5-year survival was 97%. When compared to the PAM cohort there was no difference in 5-year survival (*P* = 0.95) (Fig. 1). Recurrence-free survival at 5 years for the matched controls was 96%, and when compared to the PAM cohort there was no difference (*P* = 0.86) (Fig. 2). Of the recurrences in the control group, 25% were locoregional.

In our cohort, 3 LSD patients had a prior non-PAM ESD diagnosis which recurred during pregnancy. One patient had prior T2a disease which recurred within 1 year during subsequent pregnancy and the two others had T1a disease which recurred at 1.3 years and 2.6 years during subsequent pregnancy. No recurrences were noted in patients further than 2.6 years from their previous diagnosis. Overall, the numbers of recurrence during pregnancy were quite low, with only 3 patients in the 18-year period at our institution. Nine patients in our ESD PAM cohort and 2 in our LSD were noted to have at least one other non-PAM primary melanoma diagnosis in their lifetime.

### Current treatment approaches in PAM

No clinical guidelines for PAM currently exist. Instead, clinical approach is largely based on limited experience, non-pregnancy-associated melanoma guidelines and treatment methods for other pregnancy-associated malignancies. Most importantly at our institution, we highlight the benefit of a multispecialty team including surgical oncology, medical oncology, radiology, radiation safety, specialty pharmacists, and maternal-fetal medicine. Our approach to PAM, both early and late stage are summarized below.

Melanocytic nevi should be monitored during pregnancy and if melanoma is suspected, an excisional biopsy under local anesthesia is considered safe, regardless of gestational status, and should be performed promptly. Physicians often use lidocaine without epinephrine as a local anesthetic in pregnant patients but lidocaine with low doses of epinephrine are generally considered safe and not teratogenic in other dermatologic surgeries [20,21]. Diagnostic biopsies should be performed for suspected PAM in a manner identical to diagnostic biopsies performed for non-PAM, for example, excision with 1 to 2 mm of normal skin to increase the chance of complete lesion removal when feasible [22]. WLE recommendations are based on current NCCN guidelines for general population melanoma and are determined by Breslow depth or thickness. Most WLEs are generally safely performed at any time during gestation and should not be postponed due to pregnancy.

For SLNB there is no current guidance on timing in PAM. As with most surgical procedures during pregnancy, surgeons prefer the first or second trimester to decrease risk of induction of pre-term labor, and consultation with maternal-fetal medicine specialists is advised. To identify sentinel nodes, Lymphazurin (isosulfan blue) is not recommended due to concern for severe allergic reactions including anaphylaxis while pregnant which increases risk to the fetus [23]. Methylene blue is contraindicated due to teratogenicity (most commonly malformations include atresia of the ileum and jejunum) [23,24]. Instead for sentinel node identification, the recommendation during pregnancy is to use pre-operative intradermal technetium-99 with imaging for mapping which can be done within the threshold safety guidelines for radiation in pregnancy. The use of technetium during pregnancy poses minimal risk to the fetus, delivering a radiation dose of <5 mGy [25]. It is well-studied that radiation doses <100 milli-grays (mGy) do not increase incidence of fetal malformations [25]. SPECT-CT imaging should be avoided due to the higher radiation dose. Consultation with the radiology team to optimize imaging and minimize effective radiation dose to the fetus is advised.

For staging PAM imaging recommendations, according to the American College of Obstetrics and Gynecologists’ Committee on Obstetric practice, the techniques of choice during pregnancy include ultrasonography or MRI preferably without gadolinium [20]. MRI is considered safe for mother and baby in the second or third trimester and can be performed in the first trimester if clinically necessary. Nuclear medicine studies can be performed if necessary, since they are typically administered at doses that have not demonstrated fetal harm [20]. PET/MR can be a helpful choice for proper staging when stage IV disease is suspected as this is associated with less radiation than a CT scan of the chest, abdomen and pelvis, used less FDG than a PET-CT, and a brain MRI without gadolinium can be done simultaneously. Coordination with the multispecialty team is important as adjunctive techniques such as having the patient void immediately before getting on the scanner and keeping the patient well-hydrated minimize fetal exposure. For late-stage PAM patients, the data is even more limited in terms of treatment. Over the past decade, immune checkpoint inhibitors (ICI) and TT (BRAF/MEK inhibitors) have become the main efficacious systemic therapies for melanoma. Based on their mechanisms of action, they can cause fetal harm when administered to a pregnant woman, though human data is lacking. Therefore, these medications are often avoided during pregnancy and the oncologic and pregnancy-associated outcomes with these treatments remains largely unknown. It is known that PD-1 inhibitors can cross the placenta. BRAF inhibition with vemurafenib, without MEK inhibition, can be utilized as there is limited data that this drug does not cross the placenta and therefore might be used to treat pregnant women with stage IV disease prior to the time of safe induction of labor at 34 weeks or when deemed safe by maternal-fetal medicine specialist. There does exist in the literature, a few case reports which have reported favorable outcomes while receiving ICI and TT while pregnant including an NCI paper documenting their experience with 9 women who became pregnant while on ICI therapy in the context of clinical trial participation [21,26,27]. In terms of fetal and placental melanoma metastasis, events overall are rare [28]. However, a post-partum fetal melanoma diagnosis carries high mortality risk [29]. Thus, all currently pregnant late-stage PAM patients should have their placentas evaluated postpartum histologically and their babies should be closely monitored for the first 2 years post-partum for signs of melanoma.

## Discussion

We present what is to the best of our knowledge, data on the largest single-institution cohort of PAM patients. We found no significant difference in outcomes for PAM and non-PAM melanoma patients. We reviewed treatment details and show that there was very little deviation from SOC for our PAM cohort compared to non-PAM patients. Delays were noted at the maximum of 4 weeks and a few patients chose to not pursue SOC, similar to what is seen for non-PAM patients. This shows that the standard of care of counseling, treatment and monitoring of PAM patients can be similar to what is provided to non-PAM patients. This does require a broad team of specialists including surgical oncology, medical oncology, radiology, radiation safety, specialty pharmacists, and maternal-fetal medicine.

Our results conflict with some prior studies. Per Kyrgidis *et al*., PAM was associated with a 17% higher mortality compared with non-pregnant counterparts [14]. A meta-analysis by Byron *et al*. showed increased mortality (hazard ratio of 1.64) for PAM compared to melanoma, however, the including studies had many varying definition for PAM and thus included non-consistent populations [13]. Moller *et al*., used England’s cancer registry from 1998 to 2007 to report an age-adjusted HR of 2.06 (1.42–3.01) for women diagnosed with melanoma within the year after they gave birth (306 patients) [30]. Stensheim *et al*. reported a 50% increase in mortality in women diagnosed with melanoma during pregnancy using 160 patients from a Norway cancer registry [31]. Worse PAM outcomes have been theoretically multifactorial in the setting of PAM hormonal drivers, compromised host immune status, increased cellular tolerance through T cell variation, increased lymphangiogenesis in the setting of pregnancy and delays in diagnostic and therapeutic management due to patient and provider preferences [13–16]. We also suspect that the wide variety of populations and institutions included in meta-analysis may have led to worsening outcomes. Surgical and medical treatments have also advanced since this data was published.

A number of other studies, like our results, directly contradict these findings and suggest that outcomes for PAM are not worse than for non-PAM patients, regardless of stage [17–19,32]. The largest population-based cohort currently published includes 1019 Swedish women diagnosed with melanoma during pregnancy or within 2 years of childbirth between 1965 and 2009 which reports an age-adjusted HR of 1.09 (0.83–1.42) [33]. These data are in line with our findings, but report on outcomes prior to the current practice of primary and adjuvant systemic therapy with targeted and immunotherapies.

In terms of other PAM characteristics previously reported, de Haan *et al*. had previously reported a high percentage of late-stage disease (50% stage III and IV) in a 60-patient PAM cohort they collected using the International Network on Cancer, Infertility and Pregnancy. Our cohort, however, was 81.9% stage I and II disease at the time of PAM diagnosis [34]. Jones *et al*. noted an association between PAM diagnosis and increased parity [17]. Interestingly, this is also contradicted by study with Lambe *et al*. who noted that early pregnancy and increased parity lowered a patient’s risk for PAM [35]. We, however, show the average parity of our cohort is only 1.2 and found no difference in parity between the ESD and LSD PAM cohorts. We also note that the average BMI of our patients at the time of diagnosis is 21.8 kg/m^2^ which makes excess estrogen due to increased adiposity less likely to be associated with increased rates of PAM. As reported in the Swedish cohort study, the average age of women in our PAM cohort is 31 years and the anatomic site of the majority of primary lesions was the trunk [33].

The risk of melanoma recurrence during pregnancy is another difficult issue facing women after treatment for melanoma. There are no guidelines for patients or providers in terms of preconception counseling for these patients. There has been no evidence to date to suggest that a post-cancer pregnancy worsens prognosis in these patients [36,37]. Mackie *et al*. note that initial melanoma thickness was the best indicator for increased risk of recurrence and recommended a 2 year waiting period from cancer treatment to pregnancy based on their data that 83% of patients that experienced recurrence with stage II disease do so in the first 2 years [38]. Others suggest waiting time closer to 3–5 years based on presumed individual risk of reoccurrence without much supporting data. Overall, the numbers of recurrence during pregnancy were quite low, only 3 patients. Nine patients in our ESD PAM cohort and 2 in our LSD were noted to have at least one other non-PAM primary melanoma diagnosis in their lifetime. Clearly, patients with prior melanoma diagnosis are at increased risk of future melanoma diagnoses as well as PAM. This supports close skin examination of patients considering pregnancy and throughout their term for those with any prior melanoma diagnosis. We also suggest that women with prior non-PAM history, patient education and consultation with oncology should be considered prior to getting pregnant.

Compared to larger registry-based studies, our data is from a well-maintained and reliable single institution database with extensive follow-up further validated by granular medical record review. However, our study includes treatment-related and population biases inherent to our highly specialized cancer care referral center. Notably, our study population lacks racial diversity, which limits its generalizability to other populations. However, melanoma does have a known higher incidence in the white population in general. Overall, the lifetime risk of getting melanoma is about 2.6% (1 in 38) for whites, 0.1% (1 in 1000) for Blacks, and 0.6% (1 in 167) for Hispanics [39]. We also recognize that pregnancy typically increases a patient’s contact time with providers. Some of the melanoma lesions identified during pregnancy may have been pre-existing and incidentally discovered during prenatal visits.

The outcomes of PAM following standard-of-care treatment at a highly specialized center for melanoma care were comparable to non-PAM when matched by clinical-pathological features. This is likely explained by benefit of a multispecialty team including surgical oncology, medical oncology, radiology, radiation safety, specialty pharmacists, and maternal-fetal medicine. More published data will allow depth of understanding and nuance needed to create PAM treatment guidelines.

## Acknowledgements

Grant funding through NCI CA090628.

### Conflicts of interest

There are no conflicts of interest.
